# Analysis of trends in the context of implant therapy in a university surgical specialty clinic: a 20-year retrospective study

**DOI:** 10.1007/s00784-024-06033-2

**Published:** 2024-12-23

**Authors:** Clemens Raabe, Emilio Couso-Queiruga, Jennifer Tjokro, Daniel Buser, Michael M. Bornstein, Manrique Fonseca, Frank Schwarz, Vivianne Chappuis

**Affiliations:** 1 Department of Oral Surgery and Stomatology, School of Dental Medicine, University of Bern, Bern, Switzerland; 2 School of Dental Medicine, University of Bern, Bern, Switzerland; 3 Department of Oral Health & Medicine, University Center for Dental Medicine Basel UZB, University of Basel, Basel, Switzerland; 4 Department of Reconstructive Dentistry and Gerodontology, School of Dental Medicine, University of Bern, Bern, Switzerland; 5 Department of Oral Surgery and Implantology, Goethe University, Carolinum, Frankfurt am Main, Germany

**Keywords:** Dental implants, Analyses, Demographic, Bone grafting, Sinus floor augmentation, Guided bone regeneration

## Abstract

**Objectives:**

To analyze the trends in the context of implant therapy in a 3-year patient population and compare it with data obtained over the last 20 years.

**Materials and methods:**

All adult subjects who received treatment in the context of implant therapy between 2020 and 2022 were included in this retrospective study. Data regarding patient demographics, indications and location of implant therapy, implant characteristics, surgical techniques, complications, and early implant failures were recorded and compared to data obtained in the years 2002–2004, 2008–2010, and 2014–2016.

**Results:**

Between 2020 and 2022, *n* = 1555 implants were placed in *n* = 1021 patients. The mean age at implant placement was 59.9 + 15.1 years, demonstrating an increase over time in the age group 61–80 years of 23.1% and > 80 years of 3.2% (*p* < 0.0001). Single tooth gaps (48.9%) remained the main indication. The use of narrow diameters ≤ 3.5 mm increased (9.4% vs. 26.6%, *p* < 0.0001), while implant lengths > 10 mm decreased (45.7% vs. 23.5%, *p* < 0.0001). A reduction in more invasive techniques and an increase in computer-assisted implant surgeries (CAIS) of 19.5% was found.

**Conclusions:**

The mean age of patients receiving dental implant therapy, with the use of narrow-diameter and shorter implants has progressively increased in the last 20 years. The observed trends suggest a transition from conventional to CAIS, accompanied by the introduction of minimally invasive surgical techniques.

**Clinical relevance:**

The adoption of narrower and shorter implants, along with minimally invasive techniques and CAIS, enables clinicians to tailor treatment plans that accommodate the unique needs of aging patients and optimize clinical outcomes.

**Supplementary Information:**

The online version contains supplementary material available at 10.1007/s00784-024-06033-2.

## Introduction

Tooth replacement therapy with dental implants has evolved into a widely established treatment option in contemporary dental practice over the last few decades, providing reliable and satisfactory long-term outcomes [1]. Concurrently, the demographics of the societies have transformed during this period, accompanied by regional variations. Industrialized nations are faced with a progressively aging population, and the substantial cohort of the baby boomer generation is entering advanced stages of life [2]. Therefore, the proportion of patients with medical risk factors, functional limitations, dependency, and frailty is increasing [3, 4]. Simultaneously, teeth in these patients are more predictably maintained in a status compatible with health, and complete edentulism rates have considerably decreased [5–7]. However, when teeth are lost, patients desire to restore their appearance, function, and quality of life to normal, expecting dental implant therapy to fulfill these needs, which has been shown in a study from Hong Kong with comparable demographics to Switzerland [8]. This is also reflected by an analysis of population trends in the U.S., which observed a significant increase in dental implant prevalence from 0.7% in 1999–2000 to 5.7% in 2015–2016, with the most pronounced growth seen among individuals aged 65 to 74 years [9].

Over the past years, dental medicine has continuously evolved with the goal of improving patient care and also integrating patient-centered outcome criteria as measures for evaluating successful treatment approaches [10]. A progressive shift from conventional, and potentially extensive, clinical procedures to the use of less invasive approaches with lower morbidity, including the help of novel digital technologies, has emerged and consolidated in daily clinical practice. In the context of implant therapy, different approaches such as alveolar ridge preservation have demonstrated their efficacy in attenuating the physiologic bone remodeling that follows unassisted socket healing [11, 12], significantly reducing the need for invasive ancillary bone augmentation procedures [13, 14]. Narrow-diameter and short dental implants have been found to provide similar or only slightly inferior survival and success rates compared to standard diameter/length implants [15–17]. They are therefore considered a reliable alternative to minimize the need for augmentation procedures in sites presenting hard tissue deficiencies, while simultaneously lowering patient morbidity. Additionally, modifications in implant micro- and macro-characteristics (e.g., deep-threaded macro designs, micro-rough surfaces with superhydrophilicity) [18, 19], advancements and incorporation of digital technologies such as 3-dimensional (3D) imaging, virtual treatment planning, and computer-assisted implant surgeries (CAIS) [5, 20, 21] have expanded potential indications for implant therapy, have helped in reducing treatment times from implant insertion to delivery of the final restoration, and have also resulted in more in-depth understanding of the planned intervention for the surgeon. These advancements are typically implemented through standardized, research-based protocols in university settings, where dental implant therapy involves comprehensive treatment planning guided by experienced specialists. This approach addresses a diverse patient population, including surgically or medically complex cases referred by general practitioners, with treatment demands influenced by demographic and epidemiological trends [5].

However, there is only scarce information on related trends in the context of implant therapy regarding type of surgical procedures or patient characteristics over time. Hence, the present study aimed to primarily analyze demographics of the implant patient pool at a surgical specialty clinic for the years 2020–2022 and compare the results to the intervals 2002–2004, 2008–2010, and 2014–2016 to identify potential changes [22–24]. The secondary aims were to analyze and compare the indications, location of therapy, implant characteristics, surgical techniques, complications, and early implant failures over these two decades. Finally, the null hypothesis was that there is no change in patient demographics (H01), indications (H02), location of therapy (H03), implant characteristics (H04), and surgical techniques (H05) over the two decades analyzed.

## Materials and methods

This retrospective study is within the continuum of a study series spanning the periods 2002–2004, 2008–2010, and 2014–2016 conducted in the Department of Oral Surgery and Stomatology at the University of Bern, Switzerland and followed the same methodology as reported in the preceding investigations [22–24]. The present retrospective study assesses anonymized health-related data from patients who gave a general consent. It was independently reviewed by the ethics committee of the state of Bern, Switzerland, which determined that it does not fall under the scope of the Human Research Act. Consequently, no formal approval was deemed necessary (ID 2023 − 01522). The study design follows the Federal Policy for the Protection of Human Subjects and is in accordance with the STROBE guidelines (Strengthening the Reporting of Observational Studies in Epidemiology) [25].

### Patient selection

This study included all records from patients who received dental implants in the Department of Oral Surgery and Stomatology, University of Bern, Switzerland, from January 2020 to December 2022. The inclusion and exclusion criteria for implant treatment were described in previous publications [26, 27]. In brief, the inclusion criteria consisted of partially and fully edentulous patients receiving dental implants with adequate bone dimensions as per implant specifications. This could include sites requiring simultaneous or staged horizontal and/or vertical bone augmentation. Exclusion criteria encompassed patients with compromised general health and local conditions contraindicating surgical intervention, such as inadequate oral hygiene, uncontrolled periodontal diseases or diabetes, immunodeficiency, high-dose anti-resorptive therapy, pregnancy, or those aged ≤ 18 years. Implants used for skeletal anchorage in orthodontic treatments, such as palatal implants or temporary anchorage devices, were not evaluated in this study.

### Clinical procedures

All implant placements were performed under local anesthesia. Antibiotic prophylaxis was prescribed two hours before surgery, based on the patient’s needs. The surgical procedures were conducted by 22 surgeons, consisting of eight senior surgeons and 14 residents specializing in oral surgery. Oversight by experienced senior surgeons ensured the quality of procedures performed by residents. Comprehensive information regarding presurgical assessments, surgical techniques, and postoperative care has been reported in previous studies [26–29]. Postoperatively, patients were prescribed oral analgesics and an antiseptic mouth rinse, unless contraindicated for medical reasons.

### Descriptive analysis

Over four months (August-December 2023), three examiners (C.R, E.C.Q, and J.T) gathered data from the patient’s records. The primary outcome variable investigated was the age of the implant patient pool for the years 2020–2022 and the comparison with the ones reported for the intervals from 2002 to 2004, 2008–2010, and 2014–2016.

The secondary outcome variables assessed include the following parameters:

- Indication for implant therapy, classified into a single-tooth gap, extended edentulous gap, distal extension, or fully edentulous jaw;
- Location of implant therapy, grouped into four regions: anterior maxilla (maxillary canine to maxillary canine), posterior maxilla (premolars and molars in the maxilla), anterior mandible (mandibular canine to mandibular canine), and posterior mandible (premolars and molars in the mandible);
- Implant characteristics, including length (in mm), diameter (in mm), design (bone-level or soft-tissue-level), and brand (e.g., Straumann, Thommen, Zeramex, Nobel Biocare);
- Surgical techniques, grouped into (1) standard implant placement (open-flap or flapless implant placement without additional bone augmentation procedures), (2) implant placement with horizontal bone augmentation (HBA, including simultaneous bone augmentation following the principles of Guided Bone Regeneration (GBR) or staged bone augmentation using GBR or autogenous bone block graft), (3) implant placement with sinus floor elevation (SFE) (either simultaneously or staged via a lateral or transcrestal approaches). Additionally, alveolar ridge preservation therapy after tooth extraction and the use of CAIS was also recorded.
- Postsurgical complications were grouped into hematoma, flap dehiscence, local signs of infection, prolonged postoperative bleeding, and temporary and permanent neurosensory disturbance. Loss of implants was recorded for early implant failures. In line with previous investigations, early implant failures were defined as implants lost during the initial healing period [22–24].

### Statistical analysis

All statistical analyses were performed with software R, version 4.10 [30]. The abovementioned variables were grouped as follows:

- Age: ≤40y, 41-60y, 61-70y, > 80y;
- Indications: single tooth gap, distal extension situation, extended edentulous gap, fully edentulous jaw;
- Location: anterior maxilla, posterior maxilla, anterior mandible, posterior mandible;
- Implant diameter: ≤3.5 mm, 3.5–4.5 mm, and > 4.5 mm;
- Implant length: ≤6 mm, > 6–8 mm, > 8–10 mm, and > 10 mm;
- Implant design: bone-level (BL), soft-tissue-level (STL);
- Surgical technique: standard implant placement, implant placement with HBA, implant placement with SFE, associated application of ARP therapy or CAIS strategies.

The data was summarized by bar plots, groups and time. Logistic regression models, involving the calculation of odds ratios (OR), were used to test for trends over time (linear in the parameter). The model’s goodness-of-fit was assessed with the help of the Hosmer-Lemeshow test. If models lacked fit, quasibinomial models were used instead. Throughout, p-values less than 0.05 were considered statistically significant (SS). P-values were variable-wisely corrected using the “Holm” method.

## Results

### Patient demographics

**Fig. 1:**
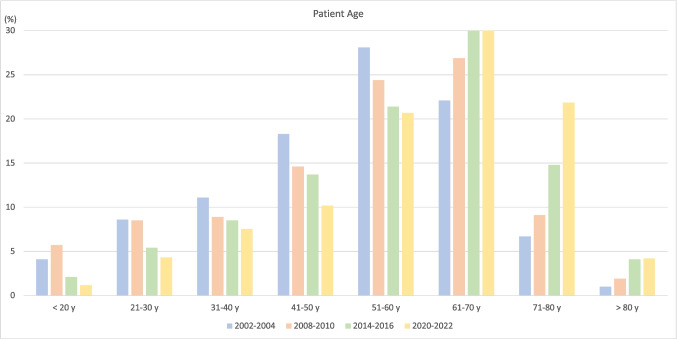
Age structure of the patient population for the periods 2002–2004, 2008–2010, 2014–2016 and 2020–2022 (RCT patients included)

The period from 2020 to 2022 included a total of 1555 implants in 1021 patients. The mean patient age at implant placement was 59.9 *±* 15.1 years (median 63 years) with a gender distribution of 50.7% female (*n* = 518) and 49.3% male (*n* = 503) (Table 1; Fig. 1). Notably, 60 patients (31 female, 29 male) with a mean age of 65.8 *±* 13.6 years, including 137 implants, were enrolled in randomized controlled trials (RCT). Information on the patient cohort excluding the RCT patients is displayed in Supplementary Tables 1 and Supplementary Fig. 1. When comparing the patient demographics for the four periods 2002–2004, 2008–2010, 2014–2016, and 2020–2022, a SS trend for a decrease in the age groups ≤ 40 years (23.8% vs. 13.0%, OR 0.96, *p* < 0.0001) and 41–60 years (46.4% vs. 30.9%, OR 0.96, *p* < 0.0001), whilst an increase in the age groups 61–80 years (28.8% vs. 51.9%, OR 1.06, *p* < 0.0001), and > 80 years (1% vs. 4.2%, OR 1.08, *p* < 0.0001) was found.

### Indications and location of implant therapy

**Fig. 2:**
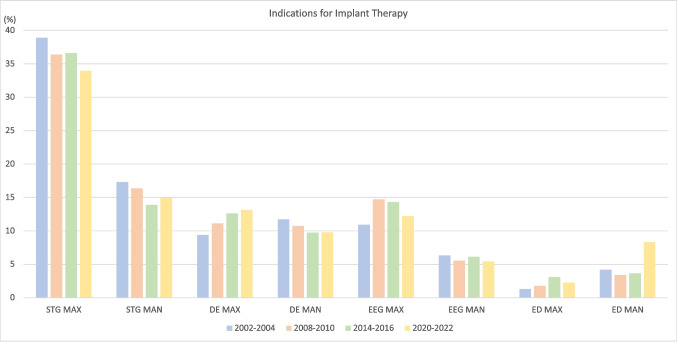
Patient distribution according to the Indication for dental implant therapy for the periods 2002–2004, 2008–2010, 2014–2016, and 2020–2022 (RCT patients included). STG: single tooth gap. DE: distal extension situation. EEG: extended edentulous gap. EJ: edentulous jaw. MAX: Maxilla. MAN: Mandible

**Fig. 3:**
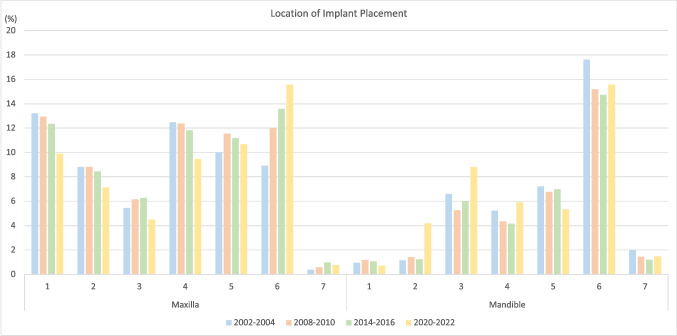
Location of dental implant placement for the periods 2002–2004, 2008–2010, 2014–2016 and 2020–2022 (RCT patients included). 1 central incisor, 2 lateral incisor, 3 canine, 4 first premolar, 5 second premolar, 6 first molar, 7 second molar

The 1021 patients presented 1105 indications for implant therapy, acknowledging instances where individual patients presented with multiple indications for dental implant placement. Single tooth gaps (48.9%, *n* = 540) were the most frequent indication, followed by distal extension (22.9%, *n* = 253), extended edentulous gaps (17.6%, *n* = 195), and fully edentulous jaws (10.6%, *n* = 117) (Table 2; Fig. 2). A larger number of implants was placed in the maxilla (58%, *n* = 903) compared to the mandible (42%, *n* = 652) (Table 3; Fig. 3). Notably, 59 indications were allocated to the RCT, including 17 posterior single tooth gaps, one posterior distal extension situation, one posterior extended edentulous gap, and 40 fully edentulous jaws in the mandible. Corresponding study implants were located at the region of the *lower lateral incisor (n = 38), canine (n = 44), first premolar (n = 36), and first molar (n = 19)*. Information on the patient cohort excluding the RCT patients is displayed in Supplementary Table 2, Supplementary Fig. 2, Supplementary Table 3, and Supplementary Fig. 3. When comparing the indications for the four periods, an SS trend for a decrease in single-tooth gaps (56.1% vs. 48.9%, OR 0.98, *p* = 0.0005) and an increase for edentulous jaws (5.6% vs. 10.6%, OR 1.04, *p* < 0.0001) was found. Regarding the implant location, a SS trend for fewer implants being placed in the anterior maxilla (27.5% vs. 21.5%, OR 0.99, *p* = 0.0006) and posterior mandibula (32% vs. 28.3%, OR 0.99, *p* = 0.01) was found, whilst an increase in the anterior mandible (8.7% vs. 13.7%, OR 1.03, *p* < 0.0001) and the posterior maxilla (31.8% vs. 36.5%, OR 1.01, *p* = 0.005) was registered.

### Implant characteristics

**Fig. 4:**
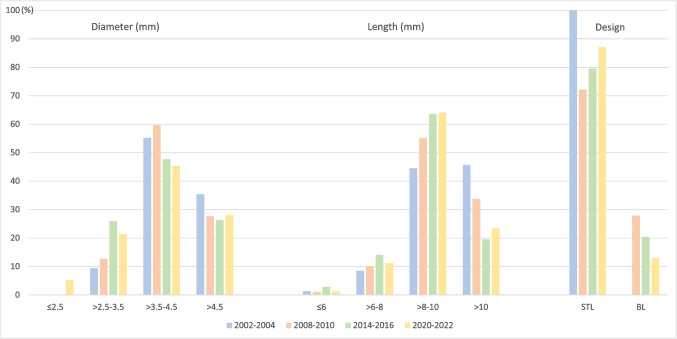
Implant characteristics for the periods 2002–2004, 2008–2010, 2014–2016 and 2020–2022 (RCT patients included). STL: soft-tissue-level. BL: bone-level

Most of the implants were manufactured by Institut Straumann AG (92.1%, *n* = 1432), followed by Thommen Medical AG (7.5%, *n* = 117), 3 M (0.3%, *n* = 4), Zeramex Dentalpoint AG (0%, *n* = 1), and Nobel Biocare AG (0%, *n* = 1). Soft-tissue-level implants were used more frequently (87.2%, *n* = 1356) compared to bone-level implants (12.8%, *n* = 199). The most common implant diameter was > 3.5–4.5 mm (45.1%, *n* = 705), followed by > 4.5 mm (28.2%, *n* = 439), > 2.5–3.5 mm (21.4%, *n* = 333) and ≤ 2.5 mm (5.2%, *n* = 81). Implant lengths included > 8–10 mm (64.1%, *n* = 997), followed by > 10 mm (23.5%, *n* = 365), > 6–8 mm (11.2%, *n* = 174), and ≤ 6 mm (1.2%, *n* = 19) (Table 4; Fig. 4). Information on the patient cohort excluding the RCT patients is displayed in Supplementary Table 4, Supplementary Fig. 4. When comparing the implant diameter for the four periods, a SS trend for an increase in implant diameters ≤ 3.5 mm (9.4% vs. 26.6%, OR 1.08, *p* < 0.0001) was found, whilst a decrease for > 3.5–4.5 mm (55.2% vs. 45.1%, OR 0.97, *p* < 0.0001) and > 4.5 mm (35.4% vs. 28.2%, OR 0.98, *p* < 0.0001) was observed. Regarding implant lengths, a SS trend for an increase in implant lengths > 6–8 mm (8.5% vs. 11.2%, OR 1.02, *p* = 0.0002) and > 8–10 mm (44.5% vs. 64.1%, OR 1.05, *p* < 0.0001) was found, whilst a decrease for implant lengths > 10 mm (45.7% vs. 23.5%, OR 0.93, *p* < 0.0001) was observed. When comparing the implant design for the periods 2008–2010 and 2020–2022, a SS trend for less use of bone-level (27.9% vs. 12.8%, OR 0.92 *p* < 0.0001) compared to tissue-level implants (72.1% vs. 87.2%, OR 1.08, *p* < 0.0001) was registered.

### Surgical techniques

**Fig. 5:**
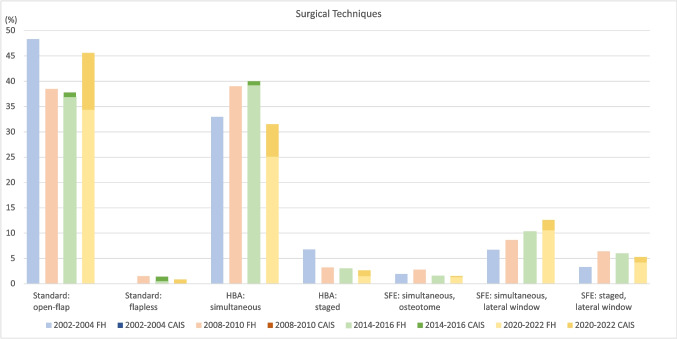
Applied surgical techniques for dental implant placement for the periods 2002–2004, 2008–2010, 2014–2016 and 2020–2022 (RCT patients included). HBA: horizontal bone augmentation. SFE: Sinus floor elevation. FH: conventional, free-handed implant placement. CAIS: computer-assisted implant surgery

Standard implant placement protocols without additional bone augmentation were applied in 46.4% (*n* = 722) of the cases, while 53.6% (*n* = 833) needed an additional bone augmentation procedure. The most frequent bone augmentation procedure was implant placement in conjunction with simultaneous HBA (31.5%, *n* = 490), followed by simultaneous SFE (14.1%, *n* = 220), staged SFE (5.3%, *n* = 82), and staged HBA procedures (2.6%, *n* = 41). Regarding SFE, the lateral approach was used in 278 (92%) cases compared to the transcrestal osteotome technique in 24 (8%) cases (Table 5; Fig. 5). Implant placement after alveolar ridge preservation was observed in 4.4% of implants (*n* = 68) and the application of guided implant placement using sCAIS was observed in 19.5% (*n* = 304) of the implant cases. Regarding implant location, horizontal bone augmentation was necessary in 60.0% of cases in anterior sites (329 of 548 implants) compared to 23.8% of cases in posterior sites (240 of 1007 implants). Flapless implant placement was limited to carefully selected cases (0.8%, *n* = 13). Information on the patient cohort excluding the RCT patients is displayed in Supplementary Table 5, Supplementary Fig. 5. When comparing the surgical techniques for the periods 2002–2004 and 2020–2022, a statistically significant trend for a decrease in HBA procedures (39.74% vs. 34.1%, OR 0.99, *p* = 0.02) and an increase in SFE (11.9% vs. 19.4%, OR 1.03, *p* < 0.0001) was found.

### Complications and early failures

The most prevalent postoperative complication was hematoma, which occurred in 11.6% of the patients (*n* = 111 patients, 163 implants). Flap dehiscence manifested in 4.4% of cases (42 patients, 55 implants). Local signs of infection were identified in 1.6% of cases (15 patients, 18 implants), and were effectively managed by local antiseptic measures using topical application of 3% hydrogen peroxide and 0.2% chlorhexidine. Prolonged postoperative bleedings were registered in 1.4% (13 patients, 24 implants), all of which were successfully addressed. Transient dysesthesia was documented in 0.4% (4 patients, 4 implants). Notably, one patient experienced permanent hypesthesia affecting the mental nerve after harvesting autogenous bone at an interforaminal mandibular donor site. Nevertheless, the patient reported no impairment due to the hypesthesia. Finally, an early implant failure rate of 0.5% was observed, affecting eight implants across seven patients. Detailed information regarding the lost implants is displayed in Table 6.

## Discussion

The present study primarily aimed to assess the demographics of a patient pool for dental implant placement for the period 2020–2022 at a surgical specialty clinic and compare the results to previous investigations following the same methodology over 20 years in the same institution. The secondary aims were to analyze changes in indications and locations of therapy, implant characteristics, surgical techniques, complications, and early implant failures for the same periods. For both primary and secondary aims, several trends were found. Therefore, H01, H02, H03, H04, and H05 were rejected.

Patient demographics showed a trend for an increasing patient age from 55.2y (2002–2004), 53.6y (2008–2010), 57.2y (2014–2016), and 59.9 years (2020–2022). Notably, this upward trajectory is primarily attributed to the age cohorts exceeding 60 years, with the most prominent growth in the cohort between 71 and 80 years. This is in line with the results from a study analyzing 7 National Health and Nutrition Examination Surveys conducted in the United States from 1999 to 2016, demonstrating the largest absolute increase of implant prevalence found in the age-group 65–74 years [9]. These age cohorts reflect the so-called baby boom period, which compared to the rest of Europe took place earlier in Switzerland reflecting a demographic development 10 years ahead of the rest of Europe [2]. With the advances in medicine, life expectancies continue to grow and are accompanied with a higher quality of life. According to the World Health Statistics 2023 from the World Health Organization, global life expectancy increased from 67 years to 73 years, from 2000 to 2019. This trend is expected to continue in the near future [31], signaling an increasing need for gerodontologically oriented treatment strategies in older patients. These strategies may prioritize surgical interventions with reduced invasiveness, such as alveolar ridge preservation, utilization of narrow-diameter implants, or CAIS. However, it is crucial to acknowledge that the period 2020–2022 corresponds to the era of the COVID-19 pandemic, marked by governmental restrictions, lockdowns, and considerable discomfort experienced by the population [32]. This is particularly pertinent to patients with increased vulnerability due to systemic factors and/or advanced age. Therefore, older age groups may be underrepresented in the present cohort when compared to the results of the periods 2002–2004, 2008–2010, and 2014–2016. A study by Feher and colleagues evaluated the patient selection and surgical procedures undertaken between March 2020 - December 2020 and compared them to pre-pandemic measures in a specialized implant clinic. Notably, they did not find an effect on patient selection and only a slight effect on surgical procedures [33].

Analysis of implant indications showed single edentulous sites as the most prominent, encompassing nearly half of all implant procedures. Nonetheless, the indications for implant placement exhibited stability across the four investigated periods when considering the patient pool exclusive of RCT participants. This observation appears to contrast with recent analyses predicting a decrease in complete edentulism in the future [5–7]. A plausible explanation may lie in heightened patient expectations regarding oral health-related quality of life and an increasing acceptance of dental implant therapy over the past few decades. In this context, implant overdentures were found to significantly enhance the quality of life compared to conventional dentures [34, 35] Additionally, it is conceivable that patients screened for the RCTs, who did not meet the inclusion criteria but opted for alternative forms of dental implant therapy, may have contributed to these findings. Interestingly, the location for implant placement remained stable, except for the upper first molar, which exhibited a consistent increase over the investigated periods [22–24]. This trend may be ascribed to the increased prevalence of SFE established over time or the alternative utilization of short dental implants.

Over recent decades, progress in implant design, materials, and surface properties has paved the way for the development of narrow-diameter implants (i.e., ≤ 3.5 mm) and short dental implants (i.e., ≤6 mm) [16, 18, 36, 37]. These innovations aim to facilitate implant placement in scenarios characterized by limited conditions such as reduced mesiodistal spaces and horizontal or vertical bone deficiencies. This trend is reflected in the present analysis, where implants with a diameter ≤ 3.5 mm demonstrate an increase in placement. Nevertheless, implants with a diameter ranging from > 3.5 to 4.5 mm, closely followed by those with a diameter exceeding 4.5 mm, still exhibit a higher prevalence nowadays. In terms of implant length, a notable decline in implants exceeding 10 mm is observed. Interestingly, implants with lengths ≤ 6 mm were exclusively used in situations of limited vertical bone availability in the posterior maxilla and mandible. However, its use was merely to avoid complex and invasive vertical bone augmentations [15, 17]. Remarkably, an upward trajectory is noted in the utilization of soft-tissue-level design implants in comparison to bone-level implants, with the latter being the preferred option mostly for single-tooth anterior esthetic area. One possible explanation may be attributed to the increasing, yet limited evidence for a smaller susceptibility for peri-implant diseases in tissue-level implants compared to bone-level implants [38, 39]. This might be an effect of the coronal relocation of the prosthetic interface, optimizing the peri-implant soft tissue adhesion, and the easier restoration due to a partly predefined emergence profile [40]. In bone-level implants, the emergence profile has been associated with a significant correlation between the contour of emergence and peri-implantitis, a relationship not identified in soft-tissue-level implants [41].

In the present investigation, the predominant surgical technique for implant placement was the standard approach involving no additional bone augmentation, with only a few instances employing a flapless approach. However, approximately one-third of implants underwent additional horizontal bone augmentation procedures, indicating a slight decline over time. This reduction might be due to the increased adoption of alveolar ridge preservation techniques during tooth extraction, which was not applied on a routine basis in the previous intervals and is reported for the first time in the present study. These therapies have shown efficacy in minimizing post-extraction dimensional changes [13]. Interestingly, only a minor portion of sites presented pronounced horizontal bone deficiencies requiring a staged approach, which may result from careful consideration of the timing of implant placement after tooth extraction [11, 12, 42, 43]. Over recent decades, there has been a trend for the simultaneous application of SFE, which was most frequently carried out using the lateral window approach. Advances in implant design like deep-threaded and/or tapered implant designs have contributed to reducing the amount of staged SFE by optimizing primary implant stability. Additionally, CAIS was found established on a broad basis in all the above-mentioned procedures, facilitating improved implant placement accuracy in demanding situations [21, 44–48]. The overall data represent the shift towards less invasive surgical techniques prioritizing reduced patient morbidity. Positive side effects of this shift include a reduction in treatment time, costs, and risks for possible complications [2]. Nevertheless, the number of early implant failures was constantly low (0.5–0.7%). This is considerably lower compared to the 1.99% as reported in a retrospective study [49].

Nonetheless, the present retrospective study has several limitations. First, the focus on a patient cohort from a single specialized university clinic, largely referred by general dentists, questions the external validity of the findings. The results may not be generalizable to other populations, as patients treated in different clinical settings may have distinct characteristics or access to varying treatment options. Second, regional variations in implant dentistry philosophies may result in other treatment approaches preferred for similar clinical situations, as surgical and reconstructive techniques can vary considerably depending on geographic location and preferred institutional protocols. These differences could influence outcomes and, consequently, limit the external validity of our findings. Third, the variability among the surgeons performing the implant surgeries within and across the study periods introduces potential bias related to personal therapeutic preferences and experience levels. Different surgeons may have different techniques, decision-making processes, and thresholds for intervention, which could influence the results of the study. Fourth, the retrospective design of this study precludes the establishment of causal relationships between confounding factors and determination of the reasons underlying clinical decision-making processes (i.e., reasons for bone augmentation procedures at the time of implant placement) in the included subjects. Further research focusing on the medical aspects of this aging patient population, along with its potential risks and implications for daily clinical practice, is warranted.

## Conclusions

Based on the limitations of the present study, it can be concluded that the mean age of patients undergoing dental implant therapy has increased over the two decades under investigation. Furthermore, there is a growing trend toward less invasive surgical techniques, narrower implant diameters (≤ 3.5 mm), and shorter implant lengths (> 6–10 mm), leading to a reduction in the need for bone augmentation procedures before or during implant placement. Additionally, an increasing number of clinical procedures are incorporating computer-assisted technologies.

## Supplementary information

Below is the link to the electronic supplementary material.

ESM1 (DOCX 507 KB)

## Data Availability

The data that support the findings of this study are available from the corresponding author upon reasonable request.

## Appendix

**Table 1:** Age structure for patient and implant populations of the periods 2002–2004, 2008–2010, 2014–2016 and 2020–2022 (RCT patients included)

Period	Age	< 20 y		21–30 y		31–40 y		41–50 y		51–60 y		61–70 y		71–80 y		> 80 y		Total	
		(n)	%	(n)	%	(n)	%	(n)	%	(n)	%	(n)	%	(n)	%	(n)	%	(n)	%
2002–2004	Women	25	2.1	54	4.5	72	6.0	107	8.9	179	14.8	149	12.4	40	3.3	7	0.6	633	52.5
	Men	24	2.0	50	4.1	62	5.1	114	9.5	160	13.3	117	9.7	41	3.4	5	0.4	573	47.5
	Total Patients	49	4.1	104	8.6	134	11.1	221	18.3	339	28	266	22.1	81	6.7	12	1.0	1206	100.0
	Implants	n/a	n/a	n/a	n/a	n/a	n/a	n/a	n/a	n/a	n/a	n/a	n/a	n/a	n/a	n/a	n/a	n/a	n/a
2008–2010	Women	47	3.0	62	4.0	77	4.9	111	7.1	197	13	219	14.0	61	3.9	18	1.1	792	50.5
	Men	42	2.7	72	4.6	62	4.0	118	7.5	186	12	203	12.9	81	5.2	12	0.8	776	49.5
	Total Patients	89	5.7	134	8.5	139	8.9	229	14.6	383	24	422	26.9	142	9.1	30	1.9	1568	100.0
	Implants	135	5.9	165	7.2	171	7.5	283	12.4	576	25	674	29.6	229	10.0	46	2.0	2279	100.0
2014–2016	Women	10	0.7	28	2.0	68	4.8	90	6.3	157	11	232	16.2	110	7.7	33	2.3	728	51.0
	Men	20	1.4	49	3.4	53	3.7	105	7.4	149	10	197	13.8	102	7.1	25	1.8	700	49.0
	Total Patients	30	2.1	77	5.4	121	8.5	195	13.7	306	21	429	30.0	212	14.8	58	4.1	1428	100.0
	Implants	44	1.9	99	4.4	160	7.1	259	11.4	468	21	736	32.6	389	17.2	106	4.7	2261	100.0
2020–2022	Women	9	0.9	24	2.4	38	3.7	46	4.5	112	11.0	152	14.9	116	11.4	21	2.1	518	50.7
	Men	3	0.3	20	2.0	39	3.8	58	5.7	99	9.7	155	15.2	107	10.5	22	2.2	503	49.3
	Total Patients	12	1.2	44	4.3	77	7.5	104	10.2	211	20.7	307	30.1	223	21.8	43	4.2	1021	100.0
	Implants	16	1.0	52	3.3	100	6.4	133	8.6	299	19.2	501	32.2	381	24.5	73	4.7	1555	100.0

**Table 2:** Indications for dental implant therapy for the periods 2002–2004, 2008–2010, 2014–2016 and 2020–2022 (RCT patients included)

Indications		2002–2004				2008–2010				2014–2016				2020–2022			
		Indications	Implants	Indications	Implants	Indications	Implants	Indications	Implants								
	Region	(n)	%	(n)	%	(n)	%	(n)	%	(n)	%	(n)	%	(n)	%	(n)	%
Single tooth gap	Maxilla	469	38.9	522	28.7	576	36.4	659	28.9	609	36.6	623	27.6	375	33.9	390	25.1
	Anterior maxilla	n/a	n/a	n/a	n/a	329	20.8	353	15.5	308	18.5	318	14.1	159	14.4	162	10.4
	Posterior maxilla	n/a	n/a	n/a	n/a	247	15.6	306	13.4	301	18.1	305	13.5	216	19.5	228	14.7
	Mandible	208	17.3	229	12.6	259	16.4	259	11.4	231	13.9	259	11.5	165	14.9	172	11.1
	Anterior mandible	n/a	n/a	n/a	n/a	28	1.8	30	1.3	27	1.6	28	1.2	11	1.0	11	0.7
	Posterior mandible	n/a	n/a	n/a	n/a	231	14.6	250	11.0	204	12.3	216	9.6	154	13.9	161	10.4
Distal extension situation	Maxilla	114	9.4	227	12.5	176	11.1	302	13.3	210	12.6	322	14.2	145	13.1	199	12.8
	Mandible	141	11.7	258	14.2	170	10.7	251	11.0	162	9.7	256	11.3	108	9.8	145	9.3
Extended edentulous gap	Maxilla	131	10.9	274	15.1	233	14.7	409	17.9	238	14.3	368	16.3	135	12.2	222	14.3
	Anterior maxilla	n/a	n/a	n/a	n/a	103	6.5	208	9.1	120	7.2	195	8.6	70	6.3	112	7.2
	Posterior maxilla	n/a	n/a	n/a	n/a	130	8.2	201	8.8	118	7.1	173	7.7	65	5.9	110	7.1
	Mandible	76	6.3	136	7.5	88	5.6	151	6.6	102	6.1	170	7.5	60	5.4	93	6.0
	Anterior mandible	n/a	n/a	n/a	n/a	17	1.1	29	1.3	21	1.3	33	1.5	14	1.3	20	1.3
	Posterior mandible	n/a	n/a	n/a	n/a	71	4.5	122	5.4	81	4.9	137	6.1	46	4.2	73	4.7
Edentulous jaw	Maxilla	16	1.3	55	3	28	1.8	98	4.3	51	3.1	148	6.5	25	2.3	92	5.9
	Mandible	51	4.2	116	6.4	54	3.4	126	5.5	61	3.7	130	5.7	92	8.3	242	15.6
	Total	1206	100.0	1817	100.0	1584	100.0	2279	100.0	1664	100.0	2261	100.0	1105	100.0	1555	100.0

**Table 3:** Location of dental implant placement according to FDI teeth numbering system for the periods 2002–2004, 2008–2010, 2014–2016 and 2020–2022 (RCT patients included)

2020–2022	(*n*)	6	121	89	70	33	50	86	68	61	37	77	77	121	6	902
	%	0.4	7.8	5.7	4.5	2.1	3.2	5.5	4.4	3.9	2.4	5.0	5.0	7.8	0.4	58.0
2014–2016	(n)	9	137	133	122	67	87	157	122	104	75	145	120	170	13	1461
	%	0.4	6.1	5.9	5.4	3.0	3.8	6.9	5.4	4.6	3.3	6.4	5.3	7.5	0.6	64.6
2008–2010	(n)	10	137	140	145	69	116	154	141	85	71	137	123	137	3	1468
	%	0.4	6.0	6.1	6.4	3.0	5.1	6.8	6.2	3.7	3.1	6.0	5.4	6.0	0.1	64.4
2002–2004	(n)	5	75	97	116	49	79	115	125	81	50	111	85	87	2	1077
	%	0.3	4.1	5.3	6.4	2.7	4.3	6.3	6.9	4.5	2.8	6.1	4.7	4.8	0.1	59.3
Maxilla FDI		17	16	15	14	13	12	11	21	22	23	24	25	26	27	Total (n)
Mandible FDI		47	46	45	44	43	42	41	31	32	33	34	35	36	37	Total (n)
2002–2004	(n)	17	151	63	52	61	11	9	8	10	59	43	68	169	19	740
	%	0.9	8.3	3.5	2.9	3.4	0.6	0.5	0.4	0.6	3.2	2.4	3.7	9.3	1.0	40.7
2008–2010	(n)	19	178	75	50	58	19	15	12	13	62	49	79	168	14	811
	%	0.8	7.8	3.3	2.2	2.5	0.8	0.7	0.5	0.6	2.7	2.2	3.5	7.4	0.6	35.6
2014–2016	(n)	18	175	83	48	73	11	14	10	17	63	46	75	158	9	800
	%	0.8	7.7	3.7	2.1	3.2	0.5	0.6	0.4	0.8	2.8	2.0	3.3	7.0	0.4	35.4
2020–2022	(n)	13	121	47	40	70	33	5	6	32	67	52	36	121	10	653
	%	0.8	7.8	3.0	2.6	4.5	2.1	0.3	0.4	2.1	4.3	3.3	2.3	7.8	0.6	42.0

**Table 4:** Implant characteristics according to implant diameter, length, and design for the patient populations for 2002–2004, 2008–2010, 2014–2016 and 2020–2022 (RCT patients included). STL: soft-tissue-level. BL: Bone-level

Implants (*n*)	Length (mm)	Diameter (mm)													Design		
Characteristics		1.8	2.4	2.9	3.3	3.5	3.75	4.0	4.1	4.5	4.8	5.0	5.5	Total	STL	BL	Total
2002–2004	≤ 6	0	0	0	0	0	0	0	7	0	18	0	0	25	25	0	25
	> 6–8	0	0	0	2	0	0	0	61	0	91	0	0	154	154	0	154
	> 8–10	0	0	0	42	0	0	0	432	0	334	0	0	808	808	0	808
	> 10	0	0	0	126	0	0	0	503	0	201	0	0	830	830	0	830
	Total	0	0	0	170	0	0	0	1003	0	644	0	0	1817	1817	0	1817
2008–2010	≤ 6	0	0	0	0	0	0	0	9	0	15	0	0	24	24	0	24
	> 6–8	0	0	0	5	0	0	0	122	0	105	0	0	232	204	28	232
	> 8–10	0	0	0	125	0	0	0	672	0	457	0	0	1254	1036	218	1254
	> 10	0	0	0	160	0	0	0	556	0	52	0	0	768	379	389	768
	Total	0	0	0	290	0	0	0	1359	0	629	0	0	2278	1643	635	2278
2014–2016	≤ 6	0	0	0	0	0	0	0	24	0	38	0	0	62	62	0	62
	> 6–8	0	0	0	62	0	0	0	110	0	132	0	0	304	286	18	304
	> 8–10	0	0	1	314	0	0	0	680	0	383	0	0	1378	1140	238	1378
	> 10	0	0	1	185	0	0	0	220	0	18	0	0	424	236	188	424
	Total	0	0	2	561	0	0	0	1034	0	5	0	0	2168	1724	444	2168
2020–2022	≤ 6	0	0	0	0	0	0	0	4	0	15	0	0	19	19	0	19
	> 6–8	0	0	0	12	0	0	4	64	8	84	2	0	174	158	16	174
	> 8–10	4	31	1	221	5	10	13	381	25	298	1	7	997	895	102	997
	> 10	0	46	3	82	9	7	47	121	18	18	12	2	365	284	81	365
	Total	4	77	4	315	14	17	64	570	51	415	15	9	1555	1356	199	1555

**Table 5:** Applied surgical techniques for dental implant placement for the periods 2002–2004, 2008–2010, 2014–2016 and 2020–2022 (RCT patients included). HBA: horizontal bone augmentation. SFE: Sinus floor elevation. FH: conventional, free-handed implant placement. CAIS: computer-assisted implant surgery

Surgical procedure	2002–2004 (*n*)	%	2008–2010 (*n*)	%	2014–2016 (*n*)	%	2020–2022 (*n*)	%
Standard implant placement	878	48.3	911	40.0	886	39.2	722	46.4
FH	878	48.3	911	40.0	846	37.4	570	36.7
CAIS	0	0.0	0	0.0	40	1.8	152	9.8
Open-flap procedures	878	48.3	877	38.5	854	37.8	709	45.6
Flapless procedures	0	0.0	34	1.5	32	1.4	13	0.8
lmplant placement with HBA	722	39.7	962	42.2	972	43.0	531	34.1
FH	722	39.7	962	42.2	955	42.2	429	27.6
CAIS	0	0.0	0	0.0	17	0.8	102	6.6
Simultaneous HBA	599	33.0	889	39.0	904	40.0	490	31.5
Staged HBA	123	6.8	73	3.2	68	3.0	41	2.6
lmplant placement with SFE	217	11.9	406	17.8	403	17.8	302	19.4
FH	217	11.9	406	17.8	403	17.8	252	16.2
CAIS	0	0.0	0	0.0	0	0.0	50	3.2
Simultaneous osteotome technique	35	1.9	63	2.8	35	1.5	24	1.5
Simultaneous window technique	122	6.7	197	8.6	233	10.3	196	12.6
Staged window technique	60	3.3	146	6.4	135	6.0	82	5.3
Bone augmentation procedures (HBA + SFE)	939	51.7	1368	60.0	1375	60.8	833	53.6
Total	1817	100.0	2279	100.0	2261	100.0	1555	100.0

**Table 6:** Early implant failures in the period 2020–2022 (8 implants in 7 patients). STL: soft-tissue-level

Patient Nr.	Age	Sex	Smoking status	Implant site	Indication	Design	Diameter (mm)	Length (mm)	Augmentative procedure
1	55	m	light	35	Distal Extension	STL	4.1	10	none
2	55	m	light	36	Distal Extension	STL	4.8	10	none
3	75	m	no	11	Edentulous Jaw	STL	4.1	10	HBA
4	66	m	no	46	Distal Extension	STL	4.1	8	none
5	57	f	no	43	Edentulous Jaw	STL	3.3	12	none
6	76	f	no	16	Distal Extension	STL	4.8	8	none
7	38	m	no	46	Single Tooth Gap	STL	5.5	12	ARP
8	63	m	heavy	16	Extended edentulous space	STL	4.1	12	SFE
