# Supplementary material for: Analysis of trends in the context of implant therapy in a university surgical specialty clinic: a 20-year retrospective study
Source: Clin Oral Investig. 2024 Dec 23;29(1):27. doi: 10.1007/s00784-024-06033-2 (PMC11666676; doi:10.1007/s00784-024-06033-2)
Supplement: Supplementary file 1 — (DOCX 507 KB) [file 784_2024_6033_MOESM1_ESM.docx]

**Supplementary Tables**

|  | Age | < 20 y |  | 21-30 y |  | 31-40 y |  | 41-50 y |  | 51-60 y |  | 61-70 y |  | 71-80 y |  | > 80 y |  | Total |  |
| --- | --- | --- | --- | --- | --- | --- | --- | --- | --- | --- | --- | --- | --- | --- | --- | --- | --- | --- | --- |
|  |  | (n) | % | (n) | % | (n) | % | (n) | % | (n) | % | (n) | % | (n) | % | (n) | % | (n) | % |
| 2002-2004 | Women | 25 | 2.1 | 54 | 4.5 | 72 | 6.0 | 107 | 8.9 | 179 | 14.8 | 149 | 12.4 | 40 | 3.3 | 7 | 0.6 | 633 | 52.5 |
|  | Men | 24 | 2.0 | 50 | 4.1 | 62 | 5.1 | 114 | 9.5 | 160 | 13.3 | 117 | 9.7 | 41 | 3.4 | 5 | 0.4 | 573 | 47.5 |
|  | Total Patients | 49 | 4.1 | 104 | 8.6 | 134 | 11.1 | 221 | 18.3 | 339 | 28 | 266 | 22.1 | 81 | 6.7 | 12 | 1.0 | 1206 | 100.0 |
|  | Implants | n/a | n/a | n/a | n/a | n/a | n/a | n/a | n/a | n/a | n/a | n/a | n/a | n/a | n/a | n/a | n/a | n/a | n/a |
| 2008-2010 | Women | 47 | 3.0 | 62 | 4.0 | 77 | 4.9 | 111 | 7.1 | 197 | 13 | 219 | 14.0 | 61 | 3.9 | 18 | 1.1 | 792 | 50.5 |
|  | Men | 42 | 2.7 | 72 | 4.6 | 62 | 4.0 | 118 | 7.5 | 186 | 12 | 203 | 12.9 | 81 | 5.2 | 12 | 0.8 | 776 | 49.5 |
|  | Total Patients | 89 | 5.7 | 134 | 8.5 | 139 | 8.9 | 229 | 14.6 | 383 | 24 | 422 | 26.9 | 142 | 9.1 | 30 | 1.9 | 1568 | 100.0 |
|  | Implants | 135 | 5.9 | 165 | 7.2 | 171 | 7.5 | 283 | 12.4 | 576 | 25 | 674 | 29.6 | 229 | 10.0 | 46 | 2.0 | 2279 | 100.0 |
| 2014-2016 | Women | 10 | 0.7 | 28 | 2.0 | 68 | 4.8 | 90 | 6.3 | 157 | 11 | 232 | 16.2 | 110 | 7.7 | 33 | 2.3 | 728 | 51.0 |
|  | Men | 20 | 1.4 | 49 | 3.4 | 53 | 3.7 | 105 | 7.4 | 149 | 10 | 197 | 13.8 | 102 | 7.1 | 25 | 1.8 | 700 | 49.0 |
|  | Total Patients | 30 | 2.1 | 77 | 5.4 | 121 | 8.5 | 195 | 13.7 | 306 | 21 | 429 | 30.0 | 212 | 14.8 | 58 | 4.1 | 1428 | 100.0 |
|  | Implants | 44 | 1.9 | 99 | 4.4 | 160 | 7.1 | 259 | 11.4 | 468 | 21 | 736 | 32.6 | 389 | 17.2 | 106 | 4.7 | 2261 | 100.0 |
| 2020-2022 | Women | 9 | 0.9 | 23 | 2.4 | 36 | 3.7 | 45 | 4.7 | 104 | 10.8 | 145 | 15.1 | 107 | 11.1 | 17 | 1.8 | 486 | 50.6 |
|  | Men | 3 | 0.3 | 20 | 2.1 | 37 | 3.9 | 56 | 5.8 | 95 | 9.9 | 142 | 14.8 | 104 | 10.8 | 18 | 1.9 | 475 | 49.4 |
|  | Total Patients | 12 | 1.2 | 43 | 4.5 | 73 | 7.6 | 101 | 10.5 | 200 | 20.8 | 286 | 29.8 | 211 | 22.0 | 35 | 3.6 | 961 | 100.0 |
|  | Implants | 16 | 1.1 | 51 | 3.6 | 96 | 6.8 | 129 | 9.1 | 276 | 19.5 | 448 | 31.6 | 347 | 24.5 | 55 | 3.9 | 1418 | 100.0 |

Supplementary Table 1 Age structure for patient and implant populations of the periods 2002-2004, 2008-2010, 2014-2016 and 2020-2022 (RCT patients excluded).

| Indications |  | 2002-2004 |  |  |  | 2008-2010 |  |  |  | 2014-2016 |  |  |  | 2020-2022 | |  |  |
| --- | --- | --- | --- | --- | --- | --- | --- | --- | --- | --- | --- | --- | --- | --- | --- | --- | --- |
|  |  | Indications |  | Implants |  | Indications |  | Implants |  | Indications |  | Implants |  | Indications |  | Implants |  |
|  | Region | (n) | % | (n) | % | (n) | % | (n) | % | (n) | % | (n) | % | (n) | % | (n) | % |
| Single tooth gap | Maxilla | 469 | 38.9 | 522 | 28.7 | 576 | 36.4 | 659 | 28.9 | 609 | 36.6 | 623 | 27.6 | 375 | 35.9 | 390 | 27.5 |
|  | Anterior maxilla | n/a | n/a | n/a | n/a | 329 | 20.8 | 353 | 15.5 | 308 | 18.5 | 318 | 14.1 | 159 | 15.2 | 162 | 11.4 |
|  | Posterior maxilla | n/a | n/a | n/a | n/a | 247 | 15.6 | 306 | 13.4 | 301 | 18.1 | 305 | 13.5 | 216 | 20.7 | 228 | 16.1 |
|  | Mandible | 208 | 17.3 | 229 | 12.6 | 259 | 16.4 | 259 | 11.4 | 231 | 13.9 | 259 | 11.5 | 148 | 14.1 | 155 | 10.9 |
|  | Anterior mandible | n/a | n/a | n/a | n/a | 28 | 1.8 | 30 | 1.3 | 27 | 1.6 | 28 | 1.2 | 11 | 1.1 | 11 | 0.8 |
|  | Posterior mandible | n/a | n/a | n/a | n/a | 231 | 14.6 | 250 | 11.0 | 204 | 12.3 | 216 | 9.6 | 137 | 13.1 | 144 | 10.2 |
| Distal extension | Maxilla | 114 | 9.4 | 227 | 12.5 | 176 | 11.1 | 302 | 13.3 | 210 | 12.6 | 322 | 14.2 | 145 | 13.9 | 199 | 14.0 |
|  | Mandible | 141 | 11.7 | 258 | 14.2 | 170 | 10.7 | 251 | 11.0 | 162 | 9.7 | 256 | 11.3 | 107 | 10.2 | 144 | 10.2 |
| Extended edentulous gap | Maxilla | 131 | 10.9 | 274 | 15.1 | 233 | 14.7 | 409 | 17.9 | 238 | 14.3 | 368 | 16.3 | 135 | 12.9 | 222 | 15.7 |
|  | Anterior maxilla | n/a | n/a | n/a | n/a | 103 | 6.5 | 208 | 9.1 | 120 | 7.2 | 195 | 8.6 | 70 | 6.7 | 112 | 7.9 |
|  | Posterior maxilla | n/a | n/a | n/a | n/a | 130 | 8.2 | 201 | 8.8 | 118 | 7.1 | 173 | 7.7 | 65 | 6.2 | 110 | 7.8 |
|  | Mandible | 76 | 6.3 | 136 | 7.5 | 88 | 5.6 | 151 | 6.6 | 102 | 6.1 | 170 | 7.5 | 59 | 5.6 | 92 | 6.5 |
|  | Anterior mandible | n/a | n/a | n/a | n/a | 17 | 1.1 | 29 | 1.3 | 21 | 1.3 | 33 | 1.5 | 14 | 1.3 | 20 | 1.4 |
|  | Posterior mandible | n/a | n/a | n/a | n/a | 71 | 4.5 | 122 | 5.4 | 81 | 4.9 | 137 | 6.1 | 45 | 4.3 | 72 | 5.1 |
| Edentulous jaw | Maxilla | 16 | 1.3 | 55 | 3 | 28 | 1.8 | 98 | 4.3 | 51 | 3.1 | 148 | 6.5 | 25 | 2.4 | 92 | 6.5 |
|  | Mandible | 51 | 4.2 | 116 | 6.4 | 54 | 3.4 | 126 | 5.5 | 61 | 3.7 | 130 | 5.7 | 52 | 5.0 | 124 | 8.7 |
|  | Total | 1206 | 100.0 | 1817 | 100.0 | 1584 | 100.0 | 2279 | 100.0 | 1664 | 100.0 | 2261 | 100.0 | 1046 | 100.0 | 1418 | 100.0 |

Supplementary Table 2 Indications for dental implant therapy for the periods 2002-2004, 2008-2010, 2014-2016 and 2020-2022 (RCT patients excluded).

| 2020-2022 | (n) | 6 | 121 | 89 | 70 | 33 | 50 | 86 | 68 | 61 | 37 | 77 | 77 | 121 | 6 | 902 |
| --- | --- | --- | --- | --- | --- | --- | --- | --- | --- | --- | --- | --- | --- | --- | --- | --- |
|  | % | 0.4 | 8.5 | 6.3 | 4.9 | 2.3 | 3.5 | 6.1 | 4.8 | 4.3 | 2.6 | 5.4 | 5.4 | 8.5 | 0.4 | 63.6 |
| 2014-2016 | (n) | 9 | 137 | 133 | 122 | 67 | 87 | 157 | 122 | 104 | 75 | 145 | 120 | 170 | 13 | 1461 |
|  | % | 0.4 | 6.1 | 5.9 | 5.4 | 3.0 | 3.8 | 6.9 | 5.4 | 4.6 | 3.3 | 6.4 | 5.3 | 7.5 | 0.6 | 64.6 |
| 2008-2010 | (n) | 10 | 137 | 140 | 145 | 69 | 116 | 154 | 141 | 85 | 71 | 137 | 123 | 137 | 3 | 1468 |
|  | % | 0.4 | 6.0 | 6.1 | 6.4 | 3.0 | 5.1 | 6.8 | 6.2 | 3.7 | 3.1 | 6.0 | 5.4 | 6.0 | 0.1 | 64.4 |
| 2002-2004 | (n) | 5 | 75 | 97 | 116 | 49 | 79 | 115 | 125 | 81 | 50 | 111 | 85 | 87 | 2 | 1077 |
|  | % | 0.3 | 4.1 | 5.3 | 6.4 | 2.7 | 4.3 | 6.3 | 6.9 | 4.5 | 2.8 | 6.1 | 4.7 | 4.8 | 0.1 | 59.3 |
| Maxilla FDI |  | 17 | 16 | 15 | 14 | 13 | 12 | 11 | 21 | 22 | 23 | 24 | 25 | 26 | 27 | Total (n) |
| Mandible FDI | | 47 | 46 | 45 | 44 | 43 | 42 | 41 | 31 | 32 | 33 | 34 | 35 | 36 | 37 | Total (n) |
| 2002-2004 | (n) | 17 | 151 | 63 | 52 | 61 | 11 | 9 | 8 | 10 | 59 | 43 | 68 | 169 | 19 | 740 |
|  | % | 0.9 | 8.3 | 3.5 | 2.9 | 3.4 | 0.6 | 0.5 | 0.4 | 0.6 | 3.2 | 2.4 | 3.7 | 9.3 | 1.0 | 40.7 |
| 2008-2010 | (n) | 19 | 178 | 75 | 50 | 58 | 19 | 15 | 12 | 13 | 62 | 49 | 79 | 168 | 14 | 811 |
|  | % | 0.8 | 7.8 | 3.3 | 2.2 | 2.5 | 0.8 | 0.7 | 0.5 | 0.6 | 2.7 | 2.2 | 3.5 | 7.4 | 0.6 | 35.6 |
| 2014-2016 | (n) | 18 | 175 | 83 | 48 | 73 | 11 | 14 | 10 | 17 | 63 | 46 | 75 | 158 | 9 | 800 |
|  | % | 0.8 | 7.7 | 3.7 | 2.1 | 3.2 | 0.5 | 0.6 | 0.4 | 0.8 | 2.8 | 2.0 | 3.3 | 7.0 | 0.4 | 35.4 |
| 2020-2022 | (n) | 13 | 110 | 47 | 22 | 49 | 14 | 5 | 6 | 13 | 45 | 34 | 36 | 112 | 10 | 516 |
|  | % | 0.9 | 7.8 | 3.3 | 1.6 | 3.5 | 1.0 | 0.4 | 0.4 | 0.9 | 3.2 | 2.4 | 2.5 | 7.9 | 0.7 | 36.4 |

Supplementary Table 3 FDI-Location of dental implant placement for the periods 2002-2004, 2008-2010, 2014-2016 and 2020-2022 (RCT patients excluded).

| Implants (n) | Length (mm) | Diameter (mm) | |  |  |  |  |  |  |  |  |  |  |  | Design | |  |
| --- | --- | --- | --- | --- | --- | --- | --- | --- | --- | --- | --- | --- | --- | --- | --- | --- | --- |
| Characteristics |  | 1.8 | 2.4 | 2.9 | 3.3 | 3.5 | 3.75 | 4.0 | 4.1 | 4.5 | 4.8 | 5.0 | 5.5 | Total | Soft-tissue-level | Bone-level | Total |
| 2002-2004 | ≤6 | 0 | 0 | 0 | 0 | 0 | 0 | 0 | 7 | 0 | 18 | 0 | 0 | 25 | 25 | 0 | 25 |
|  | > 6 - 8 | 0 | 0 | 0 | 2 | 0 | 0 | 0 | 61 | 0 | 91 | 0 | 0 | 154 | 154 | 0 | 154 |
|  | > 8 - 10 | 0 | 0 | 0 | 42 | 0 | 0 | 0 | 432 | 0 | 334 | 0 | 0 | 808 | 808 | 0 | 808 |
|  | > 10 | 0 | 0 | 0 | 126 | 0 | 0 | 0 | 503 | 0 | 201 | 0 | 0 | 830 | 830 | 0 | 830 |
|  | Total | 0 | 0 | 0 | 170 | 0 | 0 | 0 | 1003 | 0 | 644 | 0 | 0 | 1817 | 1817 | 0 | 1817 |
| 2008-2010 | ≤6 | 0 | 0 | 0 | 0 | 0 | 0 | 0 | 9 | 0 | 15 | 0 | 0 | 24 | 24 | 0 | 24 |
|  | > 6 - 8 | 0 | 0 | 0 | 5 | 0 | 0 | 0 | 122 | 0 | 105 | 0 | 0 | 232 | 204 | 28 | 232 |
|  | > 8 - 10 | 0 | 0 | 0 | 125 | 0 | 0 | 0 | 672 | 0 | 457 | 0 | 0 | 1254 | 1036 | 218 | 1254 |
|  | > 10 | 0 | 0 | 0 | 160 | 0 | 0 | 0 | 556 | 0 | 52 | 0 | 0 | 768 | 379 | 389 | 768 |
|  | Total | 0 | 0 | 0 | 290 | 0 | 0 | 0 | 1359 | 0 | 629 | 0 | 0 | 2278 | 1643 | 635 | 2278 |
| 2014-2016 | ≤6 | 0 | 0 | 0 | 0 | 0 | 0 | 0 | 24 | 0 | 38 | 0 | 0 | 62 | 62 | 0 | 62 |
|  | > 6 - 8 | 0 | 0 | 0 | 62 | 0 | 0 | 0 | 110 | 0 | 132 | 0 | 0 | 304 | 286 | 18 | 304 |
|  | > 8 - 10 | 0 | 0 | 1 | 314 | 0 | 0 | 0 | 680 | 0 | 383 | 0 | 0 | 1378 | 1140 | 238 | 1378 |
|  | > 10 | 0 | 0 | 1 | 185 | 0 | 0 | 0 | 220 | 0 | 18 | 0 | 0 | 424 | 236 | 188 | 424 |
|  | Total | 0 | 0 | 2 | 561 | 0 | 0 | 0 | 1034 | 0 | 5 | 0 | 0 | 2168 | 1724 | 444 | 2168 |
| 2020-2022 | ≤6 | 0 | 0 | 0 | 0 | 0 | 0 | 0 | 4 | 0 | 15 | 0 | 0 | 19 | 19 | 0 | 19 |
|  | > 6 - 8 | 0 | 0 | 0 | 12 | 0 | 0 | 4 | 64 | 8 | 84 | 2 | 0 | 174 | 158 | 16 | 174 |
|  | > 8 - 10 | 4 | 0 | 1 | 201 | 5 | 10 | 13 | 381 | 18 | 298 | 1 | 1 | 933 | 831 | 102 | 933 |
|  | > 10 | 0 | 0 | 3 | 60 | 9 | 7 | 47 | 121 | 15 | 18 | 12 | 0 | 292 | 211 | 81 | 292 |
|  | Total | 4 | 0 | 4 | 273 | 14 | 17 | 64 | 570 | 41 | 415 | 15 | 1 | 1418 | 1219 | 199 | 1418 |

Supplementary Table 4 Implant characteristics according to implant diameter, length, and design for the patient populations for the periods 2002-2004, 2008-2010, 2014-2016 and 2020-2022 (RCT patients excluded).

| Surgical procedure | 2002-2004  (n) | % | 2008-2010  (n) | % | 2014-2016  (n) | % | 2020-2022  (n) | % |
| --- | --- | --- | --- | --- | --- | --- | --- | --- |
| Standard implant placement | 878 | 48.3 | 911 | 40.0 | 886 | 39.2 | 588 | 41.5 |
| FH | 878 | 48.3 | 911 | 40.0 | 846 | 37.4 | 452 | 31.9 |
| CAIS | 0 | 0.0 | 0 | 0.0 | 40 | 1.8 | 136 | 9.6 |
| Open-flap procedures | 878 | 48.3 | 877 | 38.5 | 854 | 37.8 | 582 | 41.0 |
| Flapless procedures | 0 | 0.0 | 34 | 1.5 | 32 | 1.4 | 6 | 0.4 |
| lmplant placement with HBA | 722 | 39.7 | 962 | 42.2 | 972 | 43.0 | 528 | 37.2 |
| FH | 722 | 39.7 | 962 | 42.2 | 955 | 42.2 | 429 | 30.3 |
| CAIS | 0 | 0.0 | 0 | 0.0 | 17 | 0.8 | 99 | 7.0 |
| Simultaneous HBA | 599 | 33.0 | 889 | 39.0 | 904 | 40.0 | 487 | 34.3 |
| Staged HBA | 123 | 6.8 | 73 | 3.2 | 68 | 3.0 | 41 | 2.9 |
| lmplant placement with SFE | 217 | 11.9 | 406 | 17.8 | 403 | 17.8 | 302 | 21.3 |
| FH | 217 | 11.9 | 406 | 17.8 | 403 | 17.8 | 252 | 17.8 |
| CAIS | 0 | 0.0 | 0 | 0.0 | 0 | 0.0 | 50 | 3.5 |
| Simultaneous osteotome technique | 35 | 1.9 | 63 | 2.8 | 35 | 1.5 | 24 | 1.7 |
| Simultaneous window technique | 122 | 6.7 | 197 | 8.6 | 233 | 10.3 | 196 | 13.8 |
| Staged window technique | 60 | 3.3 | 146 | 6.4 | 135 | 6.0 | 82 | 5.8 |
| Bone augmentation procedures (HBA + SFE) | 939 | 51.7 | 1368 | 60.0 | 1375 | 60.8 | 830 | 58.5 |
| Total | 1817 | 100.0 | 2279 | 100.0 | 2261 | 100.0 | 1418 | 100.0 |

Supplementary Table 6 Applied surgical techniques for dental implant placement for the periods 2002-2004, 2008-2010, 2014-2016 and 2020-2022 (RCT patients included). HBA: horizontal bone augmentation. SFE: Sinus floor elevation. FH: conventional, free-handed implant placement. CAIS: Computer-assisted implant surgery.

**Supplementary Figures**

Supplementary Figure 1 Age structure of the patient population for the periods 2002-2004, 2008-2010, 2014-2016 and 2020-2022 (RCT patients excluded).

Supplementary Figure 2 Patient distribution according to the Indication for dental implant therapy for the periods 2002-2004, 2008-2010, 2014-2016 and 2020-2022 (RCT patients excluded). STG: single tooth gap. DE: distal extension. EEG: extended edentulous gap. EJ: edentulous jaw. MAX: Maxilla. MAN: Mandible.

Supplementary Figure 3 Location of dental implant placement for the periods 2002-2004, 2008-2010, 2014-2016 and 2020-2022 (RCT patients excluded). 1 central incisor, 2 lateral incisor, 3 canine, 4 first premolar, 5 second premolar, 6 first molar, 7 second molar.

Supplementary Figure 4 Implant characteristics for the periods 2002-2004, 2008-2010, 2014-2016 and 2020-2022 (RCT patients excluded). STL: Soft-tissue-level. BL: Bone-level.

Supplementary Figure 5 Applied surgical techniques for dental implant placement for the periods 2002-2004, 2008-2010, 2014-2016 and 2020-2022 (RCT patients excluded). HBA: horizontal bone augmentation. SFE: Sinus floor elevation. FH: conventional, free-handed implant placement. CAIS: Computer-assisted implant surgery.
